# Pharmacoproteomic analysis reveals that metapristone (RU486 metabolite) intervenes E-cadherin and vimentin to realize cancer metastasis chemoprevention

**DOI:** 10.1038/srep22388

**Published:** 2016-03-02

**Authors:** Suhong Yu, Cuicui Yan, Xingtian Yang, Sudang He, Jian Liu, Chongtao Qin, Chuanzhong Huang, Yusheng Lu, Zhongping Tian, Lee Jia

**Affiliations:** 1 Cancer Metastasis Alert and Prevention Center, College of Chemistry, Fuzhou University, Fuzhou, 350002, China; 2 School of Integrated Traditional Chinese and Western Medicine, Fujian University of Traditional Chinese Medicine, Fuzhou Fujian, 350108, China; 3 Internal Oncology Laboratory, Fujian Provincial Key Laboratory of Translational Medicine Oncology, Fujian Provincial Cancer Hospital, Fuzhou, Fujian, 350002, China

## Abstract

Metapristone is the most predominant biological active metabolite of mifepristone, and being developed as a novel cancer metastasis chemopreventive agent by us. Despite its prominent metastasis chemopreventive effect, the underlying mechanism remains elusive. Our study, for the first time, demonstrated that metapristone had the ability to prevent breast cancer cells from migration, invasion, and interfere with their adhesion to endothelial cells. To explore the underlying mechanism of metapristone, we employed the iTRAQ technique to assess the effect of metapristone on MDA-MB-231 cells. In total, 5,145 proteins were identified, of which, 311 proteins showed significant differences in metapristone-treated cells compared to the control group (*P*-value < 0.05). Bioinformatic analysis showed many differentially expressed proteins (DEPs) functionally associated with post-translational modification, chaperones, translation, transcription, replication, signal transduction, etc. Importantly, many of the DEPs, such as E-cadherin, vimentin, TGF-β receptor I/II, smad2/3, β-catenin, caveolin, and dystroglycan were associated with TGF-β and Wnt signaling pathways, which were also linked to epithelial-to-mesenchymal transition (EMT) process. Further validation of the epithelial marker “E-caderin” and mesenchymal marker “vimetin” were carried out using immunoblot and immunofluorescence. These results have revealed a novel mechanism that metapristone-mediated metastasis chemoprevention is through intervening the EMT-related signaling pathways.

Breast cancer is the first leading cause of cancer mortality in women worldwide. Every year, above 1.3 million women are diagnosed with breast cancer and nearly 450,000 women die from it^1^. Metastasis, a process that cancer cells invade surrounding tissues and migrate to distal organs including lung, liver, brain, bone, and lymph nodes, is a major cause of mortality in breast cancer patients^2^. Therefore, development of safe and effective cancer metastasis chemopreventive agents is becoming important and badly needed. Metapristone, the most predominant biological active metabolite of mifepristone (RU486), is being developed as a novel cancer metastasis chemopreventive by us^3^,^4^.

Metapristone has received considerable attention to its anticancer activity recently. In our previous studies, we showed that metapristone produced comparable antitumor effect on several cancer cell lines. For example, metapristone induced HT-29 cells to be arrested at the G0/G1 stage, induced dose-dependent apoptosis, and interfered with adhesion of HT-29 cells to human umbilical vein endothelial cells (HUVECs) *in vitro*^3^,^4^,^5^.

Although the anticancer activity of metapristone has been exploited, its exact molecular mechanisms of actions and related pathways and targets towards cancer remain poorly understood. To get a more comprehensive understanding of metapristone functions on cancer, we employed the pharmacoproteomic analysis in the present study as we pioneered ten years ago^6^. Isobaric tags for relative and absolute quantitation (iTRAQ) technique is considered one of the most robust techniques for differential quantitative proteomic analysis^7^, which yields very small coefficients of variation in quantitative measurements^8^. Unlike gel-based proteomic method, iTRAQ exhibits much better sensitivity and allows the identification and accurate quantification of proteins from multiple samples^9^.

The epithelial–mesenchymal transition (EMT) is an important cellular process during which epithelial polarized cells become motile mesenchymal-appeared cells, which, in turn, promotes cancer cell invasion and metastasis^10^,^11^. The EMT process is very complex and controlled by various families of transcriptional regulators through different signaling pathways, including TGF-β, Wnt, MAPK, EGFR, PI3K and others^12^,^13^,^14^. Therefore, preventing cancer cells from epithelial-mesenchymal transition as well as intervening with the key proteins in EMT-related pathways is the main research objective for us to identify safe and effective cancer metastasis chemopreventives.

In the current study, we investigated the cancer metastasis chemopreventive effect of metapristone on the cell growth, migration, invasion and adhesion of MDA-MB-231 cells in *vitro*, and further explored the underlying molecular mechanism of metapristone by using an isobaric tag for relative and absolute quantitation iTRAQ combined with the tandem mass spectrometry (LC-ESI-MS/MS). We further identified differentially expressed proteins and potential signaling pathways in MDA-MB-231 cells after metapristone treatment. The findings reported in this study support our hypothesis and reveal, for the first time, a novel function for metapristone in the prevention of metastasis of breast cancer by intervening EMT-related signaling pathways.

## Results

### Effect of metapristone on cell viability

**Figure 1:**
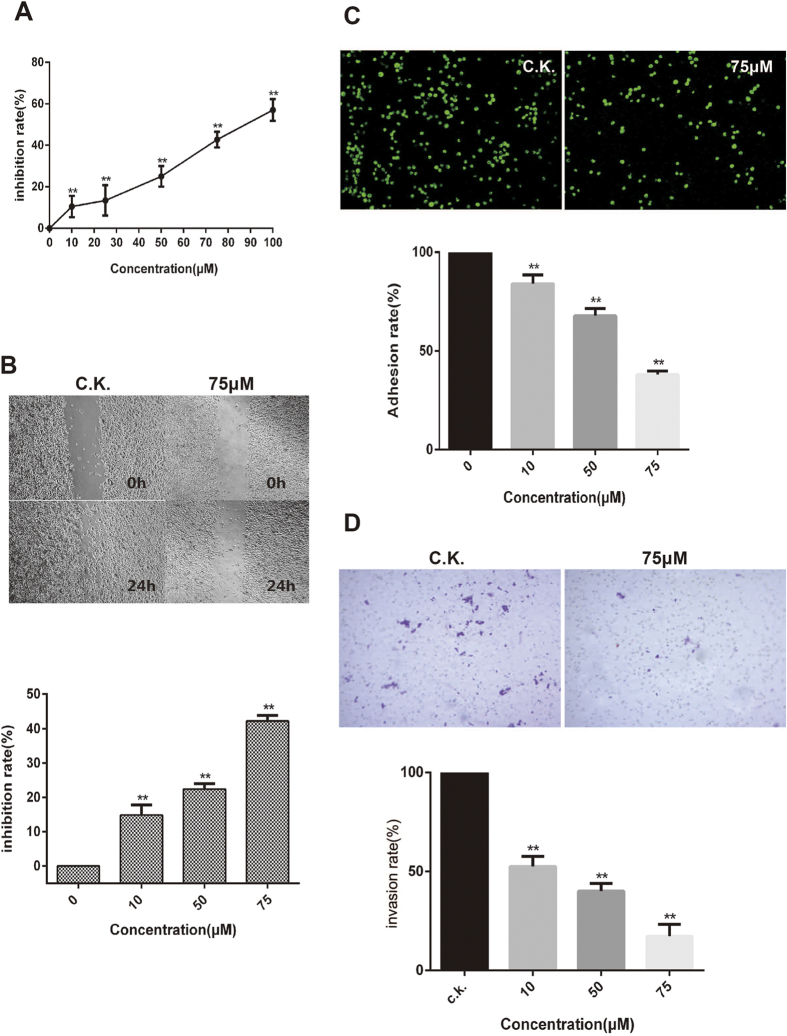
Cellular pharmacology analysis of metapristone. (**A**) *in vitro* activity of metapristone against MDA-MB-231 cell line. (**B**) dose-dependent inhibition by metapristone on cell migration. (**C**) inhibition by metapristone of MDA-MB-231 cells adhesion to HUVECs. Representative microscopic observation of the inhibition by metapristone at 0, 10, 50, and 75 μM. DMSO (0.1%) was used as vehicle control (average of 10 independent microscope fields for each of 3 independent experiments). (**D**) a Corning transwell system was used to assay cell invasion as described in methods. The amount of MDA-MB-231 cells invading through polycarbonate membranes was counted by microscopic observation (10×). Each experiment was carried out at least three times. **P < 0.01.

To explore the metastasis chempreventives function of metapristone, the cytostatic effect was examined first on human breast cancer cells MDA-MB-231 after treatment with various concentrations of metapristone for 24 h. As showed in Fig. 1A, the cytotoxicity of metapristone was low. The IC_50_ value for metapristone to suppress MDA-MB-231 cell proliferation is 91 μM.

### Metapristone inhibits cell migration, adhesion, and invasion

Would healing assay was conducted with MDA-MB-231 cells to examine the effect of metapristone on cell motility. As shown in Fig. 1B, cellular migration was controlled in a concentration-dependent manner by metapristone, being inhibited by up to 15%, 23% and 43% at 10, 50 and 75 μM, respectively (*P* < 0.01). Metapristone inhibited cell motility and wound closure at concentrations lower than its IC_50_, suggesting its specific inhibition on cell migration.

Tumor cells adhesion to the ECM is a fundamental step in cancer metastasis, the adherence of MDA-MB-231 cells to HUVECs was assessed to determine whether metapristone can regulate cell adhesion at a non-cytotoxic concentration. Ten fields of each well were randomly selected, and the adhered spots were counted. Compared with the control, the adhesion rate of MDA-MB-231 cells was 84, 68 and 39%, respectively, at 10, 50 and 75 μM of metapristone (Fig. 1C). Metapristone markedly and in a concentration-dependent manner inhibited the adherence of MDA-MB-231 cells to endothelial monolayers, indicating that it may fit into a new class of therapy for the reduction of risk factors of cancer metastasis.

It is well known that MDA-MB-231 cells have strong invasion properties in matrigel. In this study, we investigate the inhibitory effect of metapristone on cell invasion using a transwell system coated with matrigel. We found that treatment with metapristone for 24 h significantly inhibited MDA-MB-231 cells invasion through the transwell membrane. When metapristone was added at 10, 50 and 75 μM, the inhibitory effects were much more obvious compared to that of untreated group, with the inhibition rate of 48.52%, 60.06% and 82.88%, respectively (Fig. 1D).

### Overview of quantitative proteomics

**Figure 2:**
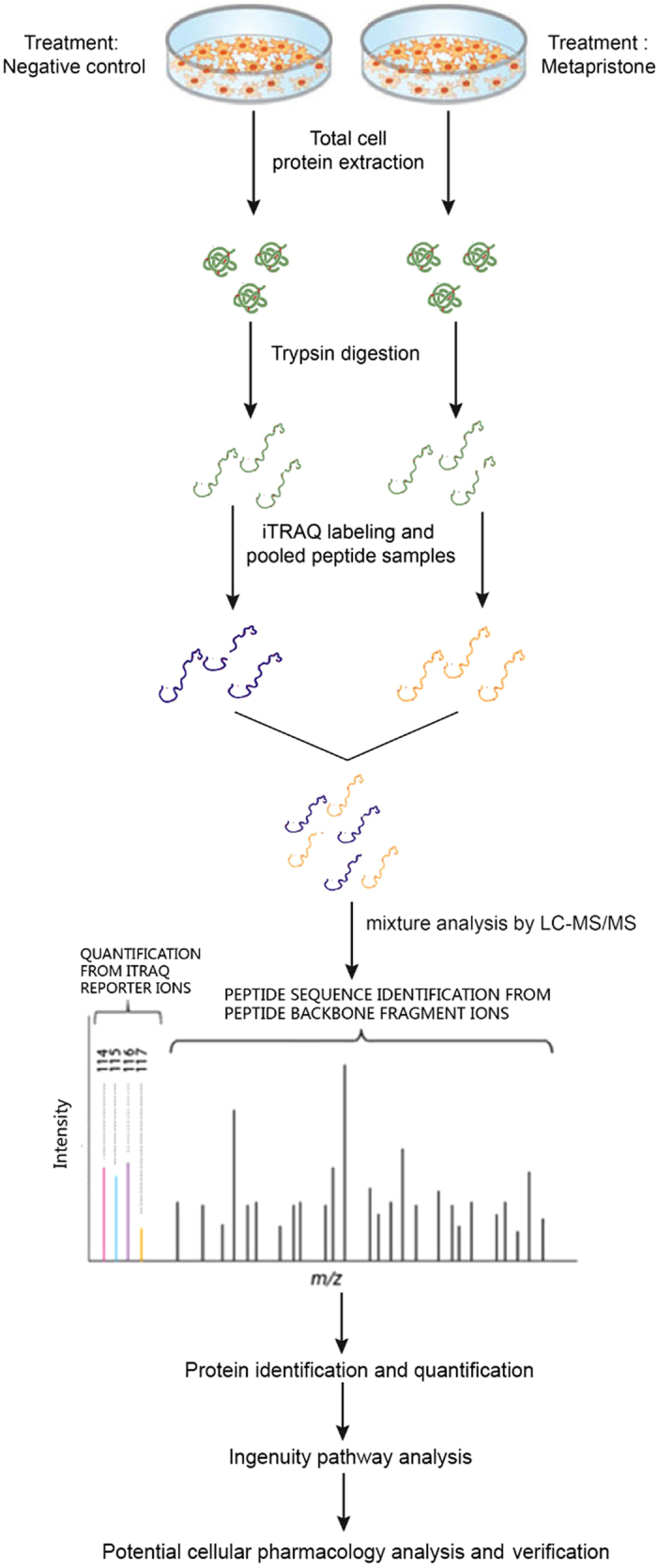
Workflow used to study differential expressed proteins in MDA-MB-231 cells after metapristone treatment using iTRAQ technology.

**Figure 3:**
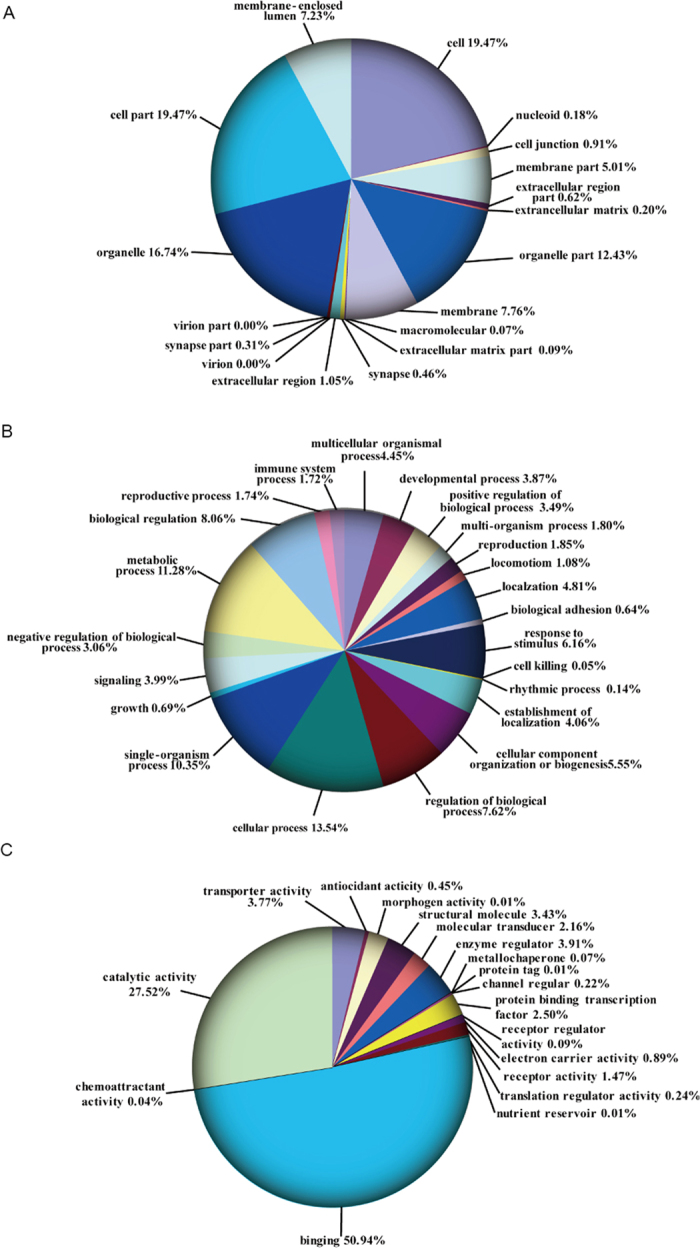
Categorization of all differential expressed proteins by GO analysis. (**A**) cellular component. (**B**) biological process. C,molecular function (*P* < 0.05).

**Figure 4:**
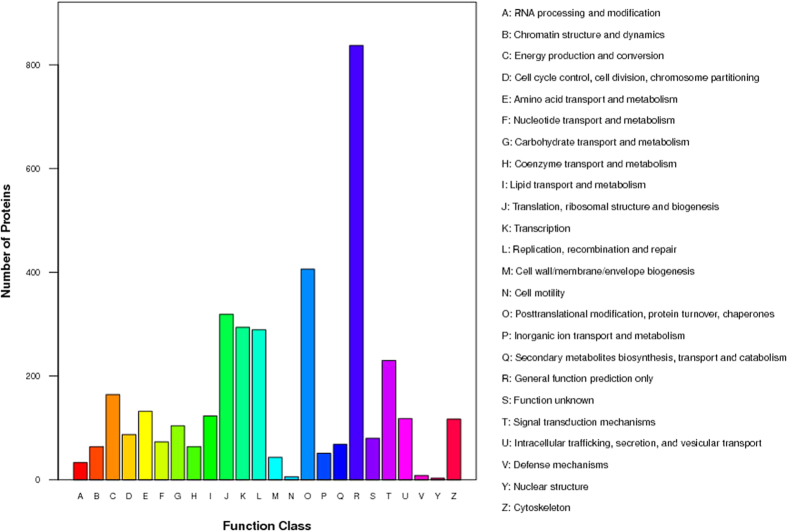
Functional category coverage of the proteins identified.

The iTRAQ analysis was performed on the purified protein extracts from MDA-MB-231 cells with or without metapristone treatment to understand the mechanism of metapristone-mediated anti-metastasis mechanism on the cellular and molecular level (Fig. 2). In total, 440,119 spectra were obtained from the iTRAQ-LC-MS/MS proteomic analysis. After data filtering to eliminate low-scoring spectra, a total of 93,114 unique spectra that met the strict confidence criteria for identification were matched to 5,145 unique proteins, of which, 311 proteins showed significant differences in metapristone-treated cells (*P*-value < 0.05). The detailed information including protein accession number, identified peptide number, protein score, sequence coverage, and regulation (fold change) for these identified proteins is shown in Table 1 and 2. Among these differentially expressed proteins (DEPs), 163 proteins were up-regulated (Table 1) and 148 proteins were down-regulated (Table 2). Then, GO analysis was conducted with the GSEABase package from R (http://www.r-project.org/) statistical platform^15^. Genes were classified in three major groups: the biological process, cellular component, and molecular function (Fig. 3A–C). Approximately 50.94% of the altered proteins were binding proteins, 27.52% were catalytic and 3.91% were enzyme regulators. In addition, we performed COG function prediction and classified these 311 positive proteins into 18 functional categories (Fig. 4).

KEGG pathway analysis was also performed based on the 311 DEPs. A total of 249 metapristone-related pathways were identified, which were assigned into 33 statistically remarkable categories (*P* value < 0.01) (Table 3), including metabolic (such as “NADH dehydrogenase”, P56181-2), Oxidative phosphorylation (such as “ATP synthase”, O75947), MAPK signaling pathway (such as “Rac GTPase activating protein 1”, B2RE34), Wnt signaling pathway (such as “RhoA”, P61586), Focal adhesion (such as “Integrin alpha-2”, P17301), ECM-receptor interaction (such as “Dystroglycan”, Q14118), VEGF signaling pathway (such as “Protein kinase C”, Q2TSD3), and TGF-beta signaling pathway (such as “TGF-beta receptor type-2”, P37173-2).

### Western blot validation of the proteomics analysis

**Figure 5:**
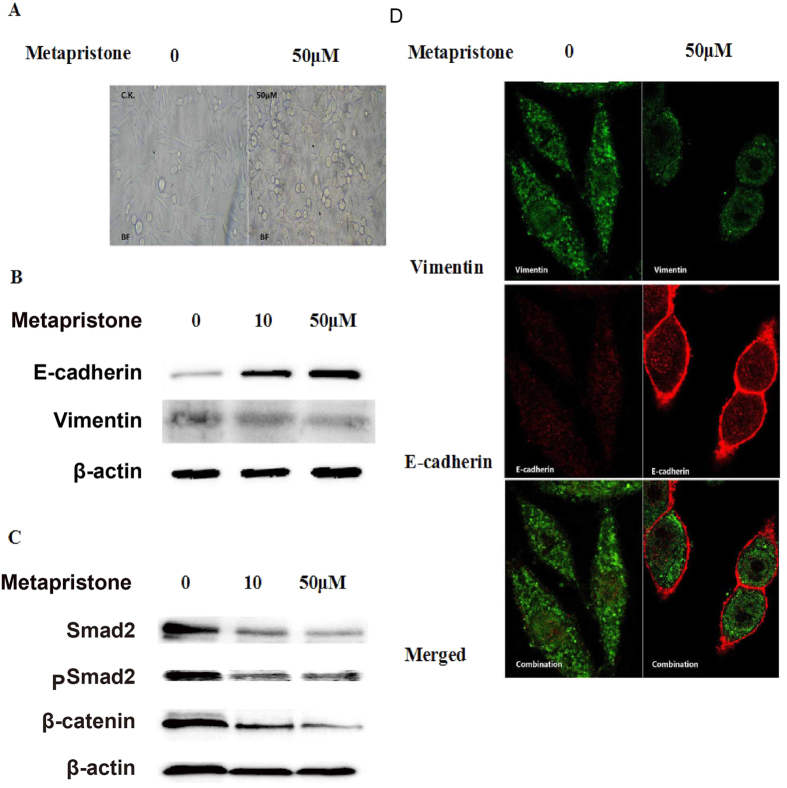
The effect of metapristone on cell morphology and EMT markers in MDA-MB-231 cells. (**A**) morphological changes were observed by phase-contrast microscopy. (**B**,**C**) the expression of vimentin E-cadherin, β-catenin, Smad2, and pSmad2 in MDA-MB-231 cells treated with or without metapristone (50 μM) was assessed by immunoblotting analysis. (**D**) confocal microscope images of vimentin immunostained with goat anti-rabbit IgG-CY3 antibody (green) and E-cadherin immunostained with goat anti-mouse IgG-FITC antibody (red) in MDA-MB-231 cells untreated or treated metapristone (50 μM).

Following the database search and classification of proteins, many differentially expressed proteins were reported to be involved in epithelial-to-mesenchymal transition (EMT), such as E-cadherin, vimentin, syndecan-1, β-catenin, dystroglycan, Smad2/3, glutaredoxin, TGF-β receptor, and so on. Western blots were performed on some selected proteins (E-cadherin, vimentin, β-catenin, and Smad 2) to further verify the iTRAQ results (Fig. 5B,C). While vimentin, one mesenchymal cell marker, was down-regulated by metapristone treatment, E-cadherin, one epithelial cell marker, strengthened with the increasing concentration of metapristone. Moreover, the expression of phosphorylation of Smad 2 was also found to be decreased by metapristone treatment. Notably, the western blot images correlated very well and thus confirmed the iTRAQ data obtained.

### Metapristone impedes EMT in MDA-MB-231cells *in vitro*

Epithelial to mesenchymal transition (EMT) and extracellular matrix degradation are critical for the initiation and progression of tumor invasion. As shown in Fig. 5A, MDA-MB-231 cells initially exhibited a typical mesenchymal-like morphology with long and narrow stretch, while cells under the treatment of metapristone showed epithelial-like morphology with relatively round extension on the plastic surface. Furthermore, we sought to determine whether metapristone could inhibit Epithelial-mesenchymal transition by regulating EMT-related markers, such as vimentin (mesenchymal-specific marker) and E-cadherin (epithelial-specific marker). As shown in Fig. 5D, up-regulated E-cadherin accumulated in the cell to cell junctions after metapristone treatment. Accordingly, the significantly reduced expression of the mesenchymal-specific marker vimentin was observed in the presence of metapristone.

## Discussion

Breast cancer metastasis accounts for the lethality of the disease and therefore there is an urgent need to develop new chemopreventives to inhibit cancer cell metastasis^16^,^17^. Experimental, epidemiological, and clinical data from the last three decades have each supported the hypothesis that oral contraceptive, such as mifepristone, possesses anticancer properties^18^,^19^. Then metapristone, the most predominant biological active metabolite of mifepristone, is being developed as a novel cancer metastasis chemopreventive agent by us.

Metastasis is a hallmark of cancer and the leading cause of mortality among cancer patients. The first step in metastasis is the migration of cancer cells away from the primary tumor, a process called tumor invasion^20^. Therefore, much research effortin recent years has been directed toward disruption of this step of the metastatic process^21^,^22^. In this study, we chose MDA-MB-231 cells with high metastatic potential to explore the effects of metapristone on the metastatic activity of human breast cancer cells. We showed that metapristone markedly inhibited their migratory (Fig. 1B) and invasive (Fig. 1D) abilities of MDA-MB-231 cells at low concentrations. Adhesion of cancer cells to ECM or vascular endothelium is also a crucial starting point of metastasis^23^. Here, we also found that metapristone markedly and in a concentration-dependent manner inhibited the adherence of MDA-MB-231 cells to endothelial monolayers. Collectively, these results suggested that metapristone had the ability to inhibit breast cancer cells metastasis. However, the underlying mechanism remains elusive.

Pharmacoproteomic, especially quantitative pharmacoproteomics, has been emerging as a powerful tool in cancer research, providing a unique avenue to investigate direct drug targets at a functional level^24^,^25^. Here, we have demonstrated the ability of the isobaric tags to detect and quantify differences in expression levels of proteins between metapristone-treated and untreated MDA-MB-231 cells that reflect functions associated with cancer cells metastasis. Temporal iTRAQ analysis identified 311 proteins as differentially expressed, with 163 as up-regulated (Table 1) and 148 as down-regulated (Table 2). Followed by GO analysis and KEGG pathway analysis, we established their potentially functional classification for the first time: there are 249 pathways, including metabolic, oxidative phosphorylation, p53, MAPK, Wnt, focal adhesion, VEGF, TGF-beta signaling pathways and so on (Table 3). Importantly, some of these pathways were reported to be linked to epithelial-to-mesenchymal transition (EMT) process, which was related with cancer carcinogenesis, prognosis and especially metastasis^14^,^26^.

The epithelial-to-mesenchymal transition (EMT) has been considered as the initiation process of cancer metastasis, when non-invasive and non-metastatic tumor cells lose their epithelial phenotype, acquire invasive properties, infiltrate surrounding tissues and metastasize to secondary sites^27^,^28^.Turning an epithelial cell into a mesenchymal cell requires loss of epithelial polarity, alteration in cellular architecture and acquisition of migrationcapacity^29^. It has also been described that during EMT, the epithelial cells acquire mesenchymal morphology, hence the expression of epithelial markers decreases and the expression of mesenchymal markers increases^30^,^31^. Here, we found that MDA-MB-231 cells initially exhibited a typical mesenchymal-like morphology with long and narrow stretch, while under the treatment of metapristone, cells showed epithelial-like morphology with relatively round extension on the plastic surface (Fig. 5A). We also found that metapristone-treatment resulted in decreased expression of mesenchymal marker “vimentin” and increased expression of epithelial marker “E-cadherin” in MDA-MB-231. Vimentin is a well-known metastasis marker and therapeutic target, as inhibiting vimentin function reduces the ability of cells to migrate^32^. Some anti-cancer drugs that are currently used in the clinic directly target vimentin such as “silibinin”^33^ and “withaferin A”^34^. One of the hallmarks of EMT is the functional loss of E-cadherin, which is thought to be a metastatic suppressor during tumor progression^35^. E-cadherin, encoded by the gene CDH1, is a transmembrane glycoprotein responsible for calcium-dependent cell-to-cell adhesion. E-cadherin plays a pivotal role in cadherin-catenin-cytoskeleton complexes, and it grants anti-invasive and anti-migratory properties to epithelial cells^36^,^37^. Our results suggest that metapristone inhibits cell migration, adhesion and invasion in highly metastatic human breast cancer cells, maybe in part, through the regulation of significant EMT-related markers which then leads to reversal of EMT.

Epithelial-to-mesenchymal transition, the process closely related to tumor development, is often regulated by a variety of signaling pathways and cytokines^12^,^13^,^14^,^26^. In this work, we performed KEGG pathway analysis based on the differential expressed proteins in MDA-MB-231 cells under metapristone-treated and untreated. We found some DEPs, including TGF β receptor I/II, Smad 2/3, RhoA, and Glutaredoxin, were related with Transforming Growth Factor β (TGF β) signaling pathway. TGF β signaling pathway has been characterized as an important inducer of EMT via several downstream signaling moleculars^13^. TGF β signals via formation of a heterotetrameric complex of TGF β receptor I/II (TGF β RI/RII), in which the active TGF β RII phosphorylates and activates the TGF β RI at the plasma membrane^38^,^39^. This conformational switch allows activated TGF β RI to interact with Smad2/3 through their MH2 domain. The activated type I receptor then propagates the signal to the nucleus by phosphorylating Smad 2 and Smad 3. Then, Smad2/3 can directly or indirectly regulate gene expression by controlling epigenetic processes, such as chromatin remodeling or by maintaining promoter DNA methylation, which is critical in silencing epithelial gene expression in cells that have undergone EMT^40^. Meanwhile, there exists a non-Smad pathway induced by TGF β^41^. In this non-Smad pathway, TGF β RII phosphorylates PAR6 (partitioning-defective protein 6), then inactivates the epithelial polarity complex, as well as activating of the small GTPase RhoA, which is contribute to cell invasion leading to breast cancer metastasis^42^. Furthermore, glutaredoxin (Grx), an anti-oxidant enzyme, was reported to play an important role in intervening TGF β-induced EMT process by reducing ROS generation in intracellular and suppressing the expression of mesenchymal markers^43^. Our results demonstrated that metapristone significantly inhibited the protein expression levels of TGF β RI/RII, RhoA, Smad 2/3, and up-regulated the expression level of glutaredoxin, implying that metapristone maybe in part, reverse EMT through attenuating TGFβ signaling pathway in MDA-MB-231 cells.

In addition to the TGF β signaling pathway, the Wnt signaling pathway also plays an important role in EMT^44^,^45^. Wnt pathway contributes to EMT by activating β-catenin, and then activating Snail, which in turn suppresses epithelial markers expression like E-cadherin^45^,^46^. Meanwhile, Caveolin-1 (CAV1), the principal structural protein of the cholesterol-rich plasma membrane invaginations, could induce EMT process through Wnt/β-catenin pathway to promote cancer metastasis^47^,^48^. Caveolin-1 is also an important regulator of cell polarity and directional movement^49^. The decreases in caveolin-1 expression follows classically described cellular changes associated with MET (including changes in cell morphology and expression of the E-cadherins and fibronectin)^50^. Our studies show that metapristone inhibits cell growth, and reverses EMT in conjunction with the activation of E-cadherin, and the inactivation of β-catenin and Caveolin-1 in MDA-MB-231 cells, implying that the MET potential of metapristone maybe related with Wnt signaling pathway.

**Figure 6:**
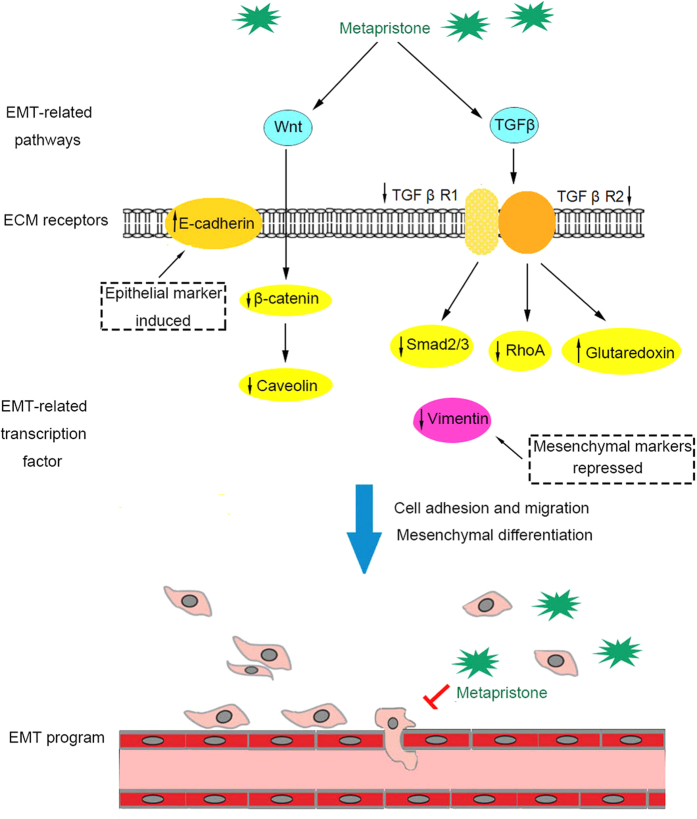
The schematic representation represents the MET potential of metapristone in MDA-MB-231 cells. Metapristone inhibits EMT by regulating TGF-β or Wnt signaling pathways. Metapristone inhibits EMT through Smad or non-Smad pathways involved in TGF-β signaling pathway, which results in suppression of mesenchymal and up-regulation of epithelial marker expression. Metapristone depressed EMT through regulating Wnt signaling pathway mediated by β-catenin and caveolin.

In conclusion, our data show that metapristone inhibits migration, adhesion, and invasion abilities of the breast cancer cells. The pharmacoproteomic study reveals that metapristone intervenes EMT-related signaling pathways, such as TGF-β and Wnt signaling pathways, in conjunction with the activation of E-cadherin and glutaredoxin and inactivation of vimentin, TGFβ RI/RII, Smad2/3, RhoA, β-catenin and Caveolin-1 (Fig. 6). These findings imply that the application of metapristone is a possible new method to control EMT, which contributes to metastatic processes in breast cancer. Our results suggest that knowledge of the putative pharmacoproteomic mechanisms will promote better use of existing drugs and facilitate the conception of new therapies and new drug development.

## Materials and Methods

### Cell culture, antibodies and reagents

MDA-MB-231 human breast cancer cells were purchased from American Type Culture Collection (ATCC, Manassas, VA) and maintained in ATCC-formulated Leibovitz’s L-15 Medium (Catalog No. 30-2008). Cells were supplemented with heat inactivated fetal bovine serum to a final concentration of 10%, and incubated at 37 °C in a free gas exchange with atmospheric air. Mouse monoclonal anti-vimentin (ab8978), -E-cadherin (ab1416), -β-actin antibodies (ab6276), goat anti-rabbit (ab150077) and goat anti-mouse (ab150115) antibodies were all obtained from Abcam Corporation.

### *In vitro* cytotoxicity studies

The cytotoxicity of metapristone was investigated by the MTT assayas described previously by this lab^51^,^52^. Briefly, MDA-MB-231 cells were seeded into 96-well plates at a density of 1 × 10^4^ cells/well, and then incubated at 37 °C in a humidified atmosphere with 100% air. After overnight incubation, the cells were treated with different concentrations of metapristone for 24 h. Culture medium was used as a blank control. Then, cells were incubation with the MTT solution (5 mg/ml) in the medium without phenol red and serum for another 4 h. The MTT-formazan formed by metabolically viable cells was dissolved in 150 μl of dimethyl sulfoxide (DMSO). Cell viability was determined by detecting the absorbance at 565 nm using an infinite M200 Pro microplate reader (Tecan, Switzerland). The absorbance of untreated cells was considered as 100%. Each sample was assayed in triplicate in three independent experiments.

### Wound healing assay

Migration of MDA-MB-231 cells was investigated in the *in vitro* wound-healing assay as described previously by this lab^3^,^51^. The MDA-MB-231 cells were seeded in 6-well plate; once confluent, 10 μg/ml mitomycin C was added. The scratch wound was generated in the surface of the plate using a pipette tip, followed by extensive washing with serum-free medium to remove cell debris. DMSO (final concentration: 0.1%) as vehicle control was added after wounding. Cells were then cultured and allowed to migrate into the wound area for up to 24 h at 37 °C. At indicated time points, motility was quantified by measuring the average extent of wound closure. Each well was counted under a light microscope (Zeiss, Germany) at a magnification of 10 × and then photographed.

### Cell invasion assay

Cell invasion assay was performed using 24-well transwells (Costar, Coring Incorporated, USA), which allows cells to migrate through a polycarbonate membrane with 8-μm pore size as we described previously^52^,^53^. Briefly, in transwell cell culture chambers, filters of 8 mm pore size were coated with Matrigel on the upper surface. MDA-MB-231 cells were resuspended with reduced serum L-15 medium and seeded 5 × 10^4^ per well on the upper chamber of the transwell apparatus. Invasion assay was performed in the presence of 0, 10, 50, 75 μM of metapristone. DMSO (final concentration: 0.1%) was used as vehicle control. After 24 h incubation, the cells on the inner layer were softly removed with a cotton swab. Then, the adherent cells on undersurface of the insert were fixed in methanol and stained with 0.1% crystal violet for 20 min. The filters were washed with PBS and images were taken by a light microscope (Zeiss, Germany) at × 200 magnification. Five fields were counted per filter in each group and the experiment was conducted in triplicate.

### Cell adhesion assay

The adhesion assay of MDA-MB-231 cells to the HUVECs was assessed according to the method described previously by this lab with minor modifications^3^,^52^. Briefly, Human umbilical vein endothelial cells (HUVECs) were isolated and utilized between passages 2 and 5, and grown to confluence in 24-well culture plates. Then, TNF-α (final concentration: 10 ng/ml) was used to activate HUVECs for 4 hours. Rhodamine 123-labled MDA-MB-231 cells were co-cultured with the HUVEC monlayers in each well, followed by treatment with metapristone for 1 hour. DMSO (0.1%) was used as the vehicle control. The nonadherent cells were removed from the plate by careful three-time washings with PBS, and the MDA-MB-231 cells bound to the HUVECs were measured by a fluorescence microscope (Zeiss, Germany). Then, ten visual fields for each well were selected randomly and taken pictures. Mean inhibition of adhesion for 10 visual fields was calculated by using the equation: % of control adhesion = [the number of adhered cells in treated group/the number of adhered cells in the control group] × 100%.

### Protein preparation and iTRAQ labeling

MDA-MB-231 cells were cultured and treated with 50 μM metapristone. Treated and untreated cells were suspended in the Lysis buffer (7 M Urea, 2 M Thiourea, 4% CHAPS, 40 mM Tris-HCl, pH8.5, 1 mM PMSF, 2 mM EDTA) and sonicated in ice. The proteins were reduced with 10 mM DTT (final concentration) at 56°C for 1 h and then alkylated by 55 mM IAM (final concentration) in the darkroom for 1 h. The reduced and alkylated protein mixtures were precipitated by adding 4 × volume of chilled acetone at −20 °C overnight. After centrifugation at 4 °C, 30000 g, the pellet was dissolved in 0.5 M TEAB (Applied Biosystems, Milan, Italy) and sonicated in ice. After centrifuging at 30000 g at 4°C, an aliquot of the supernatant was taken and protein concentration was determined using the Bradford method. Then, total protein (100 μg) of each sample was digested with Trypsin Gold (Promega, Madison, WI, USA) with the ratio of protein: trypsin = 30:1 at 37°C for 16 hours. After trypsin digestion, peptides were dried by vacuum centrifugation, reconstituted in 0.5 M TEAB, and processed according to the manufacture’s protocol for 8-plex iTRAQ reagent (Applied Biosystems, Foster City,CA). The labeled peptide mixtures were pooled and dried by vacuum centrifugation, and then fractionated using Poly SULFOETHYL ATM SCX column (200 × 4.6 mm, 5 μm particle size, 200 A° pore size) by HPLC system (Shimadzu, Japan) at flow rate 1.0 ml min-1. The eluted peptides were pooled into 20 fractions, desalted with a Strata × C 18 column, concentrated to dryness using vacuum centrifuge and then reconstituted in 0.1% formic acid for LC-MS/MS analysis.

### LC-ESI-MS/MS analysis based on Q EXACTIVE

The mass spectroscopy analysis was performed using a tandem mass spectrometry (MS/MS) in an Q EXACTIVE (Thermo Fisher Scientific, San Jose, CA) coupled online to the HPLC as described before^54^,^55^. Peptides were selected for MS/MS using high-energy collision dissociation (HCD) operating mode with a normalized collision energy setting of 27.0; ion fragments were detected in the Orbitrap at a resolution of 17500. A data-dependent procedure that alternated between one MS scan followed by 15 MS/MS scan with a following Dynamic Exclusion duration of 15s. Proteins identification was performed by using Mascot search engine (Matrix Science, London, UK; version 2.3.02). For protein quantitation, it was required that a protein contains at least two unique peptides. The quantitative protein ratios were weighted and normalized by the median ratio in Mascot. We only used ratios with *p*-values < 0.05, and only fold changes of >1.5 were considered as significant.

### Proteomic data analysis

Functional annotations of the proteins were conducted using Blast2GO program against the non-redundant protein database (NR; NCBI). The keg database (http:www.genome.jp/keg/), the COG database (http://www.ncbi.nlm.nih.gov/COG/), and GO (Gene Ontology) analyses (http://www.geneontology.org) were used to classify and group these identified proteins according to the methods reported in early literature^15^,^56^.

### Western blot analysis

Cell lysates were collected using radio immunoprecipitation (RIPA) lysis buffer, supplemented with HALT protease and phosphatase inhibitor cocktail (Thermo Scientific), and immunodetection of electrophoresis-resolved proteins was performed using standard protocols. The E-Cadherin, vimentin, Smad2, pSmad2, β-catenin, and β-actin antibodies were from Abcam. Immunodetection was accomplished using enhanced chemiluminescence, and data were acquired with a quantitative digital imaging system (Quantity One, Bio-Rad) allowing it to check for saturation. Overall emitted photons were quantified for each band, particularly for homogeneously the loading controls.

### Immunofluorescence staining and high-content confocal imaging

MDA-MB-231 cells were cultured on a 35 mm cell culture dish (NEST, GBD-35-20) to 50% confluence at least 2 days before carrying out the immunofluorescence assay. Cells (with or without metapristone-treatment) were first washed by phosphate buffer 3 times and then fixed by 4% paraformaldehyde for 30 minutes. One milliliter of 0.1% Triton-X-100 was subsequently added to culture cells for ten minutes to increase cell permeability. Cells were blocked for 30 minutes at room temperature with 10% goat serum followed by culturing with primary antibodies, mouse monoclonal anti-vimentin antibody and E-cadherin for 1 h at room temperature. Then cells were added with secondary antibodies, Goat Anti-Mouse IgG-FITC antibody (Boster, BA1101) and Goat Anti-Rabbit IgG-CY3 antibody (Boster, BA1032) respectively, and cultured in the dark for 1 h at room temperature. Phosphate buffer was used to wash cells for at least three times between every two contiguous steps. Confocal analysis was performed on a Leica-TCS-SP8 confocal microscope and the images were taken under the same parameter configuration.

### Statistical analysis

All data were analyzed using SASS software and expressed as the mean ± SD or SE. Statistical comparisons between different groups were performed using Student *t*-test. A *P* value of <0.05 was considered to be statistically significant.

## Additional Information

**How to cite this article**: Yu, S. *et al*. Pharmacoproteomic analysis reveals that metapristone (RU486 metabolite) intervenes E-cadherin and vimentin to realize cancer metastasis chemoprevention. *Sci. Rep.* **6**, 22388; doi: 10.1038/srep22388 (2016).

## Figures and Tables

**Table 1:** Annotation of up-regulated proteins after metapristone treatment in MDA-MB-231 cells.

No.	Score	% Cov	Accession number	Name	Peptides	regulation (fold change)^a^
1	226	17.3	D9HTE9	Plasma membrane citrate carrier	5	1.707*
2	503	14.5	P31040	Succinate dehydrogenase [ubiquinone] flavoprotein subunit, mitochondrial	8	3.644*
3	308	39.9	P62280	40S ribosomal protein S11	7	1.709*
4	115	19.2	Q0QEY7	Succinate dehydrogenase complex subunit B	4	3.776*
5	474	27.8	P13073	Cytochrome c oxidase subunit 4 isoform 1, mitochondrial	5	1.671*
6	267	30	Q53EW8	Thiosulfate sulfurtransferase variant	7	3.181*
7	488	30.3	E9PH29	Thioredoxin-dependent peroxide reductase, mitochondrial	6	1.538*
8	446	20.7	A2A274	Aconitate hydratase, mitochondrial	13	1.528*
9	266	24.1	Q5QNZ2	ATP synthase F(0) complex subunit B1, mitochondrial	5	1.681*
10	388	26.7	Q59FZ8	Nebulette non-muscle isoform variant	9	1.591*
11	302	14.5	A6NN80	Annexin	10	2.861*
12	410	50.3	O75947	ATP synthase subunit d, mitochondrial	7	3.498*
13	1130	34.2	Q59GB4	Dihydropyrimidinase-like 2 variant	15	1.781*
14	947	30.7	Q06210-2	Isoform 2 of Glutamine—fructose-6-phosphate aminotransferase [isomerizing] 1	16	4.479**
15	212	16.1	G3V325	Pentatricopeptide repeat-containing protein 1, mitochondrial	4	2.441*
16	2168	59.3	P00338	L-lactate dehydrogenase A chain	17	2.417*
17	135	15.3	B7Z792	cDNA FLJ53932	5	1.713*
18	518	16.9	D3DUJ0	AFG3 ATPase family gene 3-like 2, isoform CRA_a	11	1.981*
19	388	63.2	E9PN17	ATP synthase subunit g, mitochondrial	4	1.709*
20	202	25.1	Q5HYK3	2-methoxy-6-polyprenyl-1,4-benzoquinol methylase, mitochondrial	4	2.408*
21	671	45.4	P15559-2	Isoform 2 of NAD(P)H dehydrogenase [quinone] 1	9	3.405*
22	146	17.5	Q8N4T8	Carbonyl reductase family member 4	4	5.404**
23	474	29.8	P62277	40S ribosomal protein S13	5	2.403*
24	189	23.7	B2RDE0	cDNA, FLJ96567	8	6.402**
25	1329	48.2	Q53FB6	Mitochondrial aldehyde dehydrogenase 2 variant	19	5.389**
26	530	26.7	Q53FC3	Programmed cell death 6 variant	5	2.312*
27	161	20.9	Q9UK22	F-box only protein 2	5	1.975*
28	148	22.6	P18827	Syndecan-1	5	4.562*
29	458	35.2	Q5T9B7	Adenylate kinase isoenzyme 1	6	2.334*
30	237	17.5	B3KMV8	cDNA FLJ12766 fis, clone NT2RP2001520	6	3.366*
31	252	28.7	I3L1P8	Mitochondrial 2-oxoglutarate/malate carrier protein (Fragment)	6	2.364*
32	167	52.2	P56385	ATP synthase subunit e, mitochondrial	4	4.361*
33	216	24.1	B3KTJ1	cDNA FLJ38349 fis, clone FEBRA1000057	6	5.353*
34	259	22.4	Q5M7Z1	RAD23 homolog A (S. cerevisiae)	4	3.351*
35	177	30.2	Q9H479	Fructosamine-3-kinase	4	2.342*
36	160	19.5	B4DNW0	Aminoacylase-1	9	2.921*
37	775	23	O60832	H/ACA ribonucleoprotein complex subunit	10	3.339**
38	104	36.5	Q9NRV9	Heme-binding protein 1	5	1.759*
39	194	43.4	P35754	Glutaredoxin-1	4	4.368*
40	362	22	Q96I99	Succinyl-CoA ligase [GDP-forming] subunit beta, mitochondrial	9	1.837*
41	156	24.6	F5GZW3	Rho GTPase-activating protein 4	4	1.536*
42	144	33.8	A8KA74	cDNA FLJ76065	4	1.625*
43	434	24.4	B4E2Z8	cDNA FLJ61206	8	1.734*
44	904	37	P11177-2	Isoform 2 of Pyruvate dehydrogenase E1 component subunit beta, mitochondrial	10	2.333*
45	699	31.6	P00966	Argininosuccinate synthase	13	2.302*
46	109	22.6	Q5SRD1	Putative mitochondrial import inner membrane translocase subunit Tim23B	4	2.133*
47	969	25.4	Q9NSE4	Isoleucine—tRNA ligase, mitochondrial	22	6.329**
48	254	15	Q8N0 × 4	Citrate lyase subunit beta-like protein, mitochondrial	4	3.327*
49	222	15.2	Q14376	UDP-glucose 4-epimerase	4	7.325**
50	225	36.7	Q86WA8	Lon protease homolog 2, peroxisomal	4	5.324*
51	128	42.1	P43155-2	Isoform 2 of Carnitine O-acetyltransferase	5	1.724*
52	414	19.6	D7PBN3	ESRP1/RAF1 fusion protein	13	2.324*
53	2112	50.5	Q59EI9	ADP,ATP carrier protein, liver isoform T2 variant	16	7.32**
54	381	29.3	D3XNU5	E-cadherin 1	6	7.319**
55	398	22.8	D3DVA5	Rho/rac guanine nucleotide exchange factor (GEF) 2, isoform CRA_a	12	1.619*
56	346	20.8	B4E290	cDNA FLJ50039	10	8.318**
57	1299	50.5	P12429	Annexin A3	15	2.322*
58	379	22.4	B7Z3K9	Fructose-bisphosphate aldolase	8	2.315*
59	151	19.9	Q9H974-2	Isoform 2 of Queuine tRNA-ribosyltransferase subunit QTRTD1	4	1.751*
60	556	23	B4DFP1	cDNA FLJ51818	12	1.815*
61	679	45	P84085	ADP-ribosylation factor 5	8	1.663*
62	317	18.8	P80303-2	Isoform 2 of Nucleobindin-2	7	2.306*
63	402	16.5	Q8NE62	Choline dehydrogenase, mitochondrial	9	1.996*
64	1016	32.6	Q13011	Delta(3, 5)-Delta(2, 4)-dienoyl-CoA isomerase, mitochondrial	10	1.703*
65	149	36.8	Q7Z4G4-2	Isoform 2 of tRNA (guanine(10)-N2)-methyltransferase homolog	5	2.302*
66	227	21.2	B4DG80	LIM and cysteine-rich domains protein 1	5	3.271*
67	122	26.3	O00178	GTP-binding protein 1	4	2.111*
68	196	24.8	H3BNX8	Cytochrome c oxidase subunit 5A, mitochondrial	5	3.022*
69	264	31.6	O95865	N(G), N(G)-dimethylarginine dimethylaminohydrolase 2	7	2.395*
70	286	41.8	P36969-2	Isoform Cytoplasmic of Phospholipid hydroperoxide glutathione peroxidase, mitochondrial	7	1.795*
71	238	21.9	B3KTM6	Ribosomal protein L5, isoform CRA_b	5	1.694*
72	891	48.9	P18085	ADP-ribosylation factor 4	8	1.742*
73	105	18	Q96CF2	Charged multivesicular body protein 4c	4	1.562*
74	714	20.5	Q6XQN6-3	Isoform 3 of Nicotinate phosphoribosyltransferase	4	2.291*
75	419	72.1	Q9HCY8	Protein S100-A14	6	1.287*
76	416	22.7	Q8IYS1	Peptidase M20 domain-containing protein 2	9	2.286*
77	1692	35.4	Q59EK6	TNF receptor-associated protein 1 variant	21	1.986*
78	192	16.7	B3KQQ0	cDNA PSEC0007 fis, clone NT2RM1000634	8	1.784*
79	322	27.7	B4DP80	cDNA FLJ56357	6	2.283*
80	270	14.4	B4DUF1	cDNA FLJ59760	8	2.252*
81	519	28.8	P31930	Cytochrome b-c1 complex subunit 1, mitochondrial	10	3.281*
82	278	33.5	B7Z4B7	cDNA FLJ52561	7	2.218*
83	1591	37.3	K7EKE6	Lon protease homolog, mitochondrial	25	3.278*
84	345	25	P04040	Catalase	11	3.215*
85	1420	58.6	P30044-2	Isoform Cytoplasmic + peroxisomal of Peroxiredoxin-5, mitochondrial	7	2.275*
86	571	44.9	B4DNR3	cDNA FLJ52710	6	8.214**
87	213	46.8	B4DRT2	28S ribosomal protein S27, mitochondrial	4	7.212**
88	412	24.4	Q6NVY1	3-hydroxyisobutyryl-CoA hydrolase, mitochondrial	9	6.274**
89	636	47	M0R0F0	40S ribosomal protein S5 (Fragment)	10	11.271**
90	254	45.4	P61081	NEDD8-conjugating enzyme Ubc12	8	6.269**
91	524	23.1	P17858	6-phosphofructokinase, liver type	14	7.368**
92	146	11.2	Q53H22	Amidophosphoribosyltransferase	5	2.068*
93	152	19.1	P46781	40S ribosomal protein S9	4	1.968*
94	227	25.6	Q9NWV4	UPF0587 protein C1orf123	5	1.266*
95	159	19.4	B3KRI2	NADH dehydrogenase [ubiquinone] iron-sulfur protein 7, mitochondrial	4	3.214*
96	646	21.6	B2R9S4	cDNA, FLJ94534	6	1.963*
97	296	18.4	B2R6S5	Cytidylate kinase, isoform CRA_a	4	10.262**
98	576	16.1	Q9UJS0-2	Isoform 2 of Calcium-binding mitochondrial carrier protein Aralar2	8	8.261**
99	269	39.5	O75368	SH3 domain-binding glutamic acid-rich-like protein	4	7.259**
100	522	45.2	O75223	Gamma-glutamylcyclotransferase	8	6.229*
101	168	24.7	B2R9 × 3	cDNA, FLJ94599	10	5.257*
102	2592	45.5	Q6NVC0	SLC25A5 protein (Fragment) OS = Homo sapiens	16	3.255*
103	319	22.5	Q9BQ69	O-acetyl-ADP-ribose deacetylase MACROD1	6	3.154*
104	179	14.8	Q6V9R7	Solute carrier family 25 member 19	4	1.752*
105	108	23.6	Q8IW45	ATP-dependent (S)-NAD(P)H-hydrate dehydratase	5	1.922*
106	878	32.4	P35908	Keratin, type II cytoskeletal 2 epidermal	18	3.251*
107	953	37.6	P49419-2	Isoform 2 of Alpha-aminoadipic semialdehyde dehydrogenase	16	5.338**
108	751	30.1	P07384	Calpain-1 catalytic subunit	19	7.247*
109	564	62	O95336	6-phosphogluconolactonase	11	11.246**
110	176	14.1	Q9H9T3-2	Isoform 2 of Elongator complex protein 3	6	5.245*
111	2382	35.4	Q53F91	Villin 1 variant	27	3.245**
112	247	31.4	B4DP27	cDNA FLJ52153	5	2.242*
113	365	25.9	Q9NUQ9	Protein FAM49B	7	3.551*
114	268	35.6	E7EW20	Unconventional myosin-VI	9	7.241**
115	4889	43.5	B7Z2 × 9	Gamma-enolase	13	5.244*
116	494	15.2	P10253	Lysosomal alpha-glucosidase	10	3.379*
117	167	20.2	P15328	Folate receptor alphas	4	2.238*
118	166	28.4	Q13315	Serine-protein kinase ATM	6	5.238*
119	232	20.2	B3KM98	cDNA FLJ10556 fis, clone NT2RP2002479	6	6.238*
120	267	29.7	Q02338	D-beta-hydroxybutyrate dehydrogenase, mitochondrial	7	4.235*
121	673	44.2	B7Z6B8	2,4-dienoyl-CoA reductase, mitochondrial	11	9.215**
122	114	20.9	B4DQ51	Short/branched chain-specific acyl-CoA dehydrogenase, mitochondrial	4	8.234**
123	2746	43.8	P40939	Trifunctional enzyme subunit alpha, mitochondrial	31	7.212**
124	437	28.9	P31937	3-hydroxyisobutyrate dehydrogenase, mitochondrial	6	5.212*
125	214	33.5	B3KTS4	cDNA FLJ38665 fis, clone HLUNG2003378	8	4.222*
126	3670	56.3	P06899	Histone H2B type 1-J	8	10.231**
127	5762	59.3	Q13885	Tubulin beta-2A chain	21	1.831*
128	647	27.9	B2RAH7	cDNA, FLJ94921	16	1.529*
129	443	17.5	O95202	LETM1 and EF-hand domain-containing protein 1, mitochondrial	11	2.229*
130	766	24.7	Q0VGA5	SARS protein s	10	1.729*
131	407	33.7	Q9Y305-2	Isoform 2 of Acyl-coenzyme A thioesterase 9, mitochondrial	12	1.855*
132	317	35.1	P63000-2	Isoform B of Ras-related C3 botulinum toxin substrate 1	7	2.277*
133	103	21.5	Q9C0C9	Ubiquitin-conjugating enzyme E2 O	5	6.217**
134	114	14.1	P04792	Heat shock protein beta-1	4	5.246*
135	248	15.7	Q9UBF2	Coatomer subunit gamma-2	4	6.213*
136	356	63.9	P07741	Adenine phosphoribosyltransferase	8	7.273**
137	260	14.7	Q8TE67-2	Isoform 2 of Epidermal growth factor receptor kinase substrate 8-like protein 3	8	2.222*
138	2952	49	P09211	Glutathione S-transferase P	9	1.825*
139	343	43.8	Q8TCD5	5~(3~)-deoxyribonucleotidase, cytosolic type	6	2.261*
140	234	26.9	Q8NCF7	cDNA FLJ90278 fis, clone NT2RP1000325	10	2.254*
141	739	45.2	P14550	Alcohol dehydrogenase [NADP(+)]	13	1.722*
142	138	19	B2RCC2	cDNA, FLJ95978	5	1.998*
143	235	16.4	Q9BRQ8	Apoptosis-inducing factor 2	6	9.217**
144	267	39	P30046	D-dopachrome decarboxylase	4	3.217*
145	144	16.4	B4DRN7	C2 domain-containing protein 5	5	8.216**
146	1762	51.4	Q6LES2	Annexin (Fragment)	15	8.113**
147	383	21.8	Q5JNW7	Proteasome subunit beta type-8	4	12.212**
148	126	20.9	Q96GD0	Pyridoxal phosphate phosphatase	4	7.202*
149	516	45.7	Q9NQR4	Omega-amidase NIT2	11	7.254**
150	278	16.6	B3KM97	cDNA FLJ10554 fis, clone NT2RP2002385	5	1.911*
151	427	25.1	J3QQX3	NADPH:adrenodoxin oxidoreductase, mitochondrial	8	1.721*
152	184	19.1	P47929	Galectin-7	4	7.209**
153	125	22.9	Q6PJ77	BTF3L4 protein (Fragment)	4	6.209**
154	228	17.3	R4GMU1	GDH/6PGL endoplasmic bifunctional protein	9	5.248**
155	326	32.7	B2R7T6	cDNA, FLJ93596	12	7.217**
156	175	21.2	Q9BUL8	Programmed cell death protein 10	5	3.252*
157	264	19.6	B2R673	cDNA, FLJ92818	9	1.884*
158	341	34.1	G8JLB3	tRNA pseudouridine synthase (Fragment)	11	1.653*
159	1093	26	F8W930	Insulin-like growth factor 2 mRNA-binding protein 2	13	8.224**
160	169	16	Q15031	Probable leucine—tRNA ligase, mitochondrial	5	5.263*
161	2146	44.7	O43175	D-3-phosphoglycerate dehydrogenase	20	5.211*
162	516	22.2	B4E0B1	cDNA FLJ52100	4	3.202*
163	151	20.3	B4DKL4	Lipolysis-stimulated lipoprotein receptor	6	2.328*

^a^Regulations (fold-changes) of differentially expressed proteins in MDA-MB-231 cells (metapristone-treatment versus control). *P < 0.05; **P < 0.01.

**Table 2:** Annotation of down-regulated proteins after metapristone treatment in MDA-MB-231 cells.

No.	Score	% Cov	Accession number	Name	Peptides	regulation (fold change)^a^
1	701	35.3	A8K9B9	cDNA FLJ77391	17	0.331*
2	286	31.4	B3KQF5	cDNA FLJ90381 fis, clone NT2RP2005035	8	0.511*
3	451	20.1	Q9H089	Large subunit GTPase 1 homolog	11	0.621*
4	167	22.4	A7UJ17	DnaJ	4	0.431*
5	178	18.3	Q53HF3	Galactosidase, alpha variant	4	0.383*
6	165	14.7	Q9BTM9-2	Isoform 2 of Ubiquitin-related modifier 1	4	0.501*
7	263	22.7	Q8NAF0	Zinc finger protein 579	4	0.528*
8	221	18.1	Q9BPX3	Condensin complex subunit 3	10	0.438*
9	122	21.3	J3QTQ0	Dystonin	8	0.607*
10	175	16.2	B4DQM4	GPN-loop GTPase 1	4	0.527*
11	437	27	J3KQA0	Synaptotagmin I, isoform CRA_b	10	0.694*
12	119	15.4	B2R728	cDNA, FLJ9325	4	0.603*
13	133	23.1	Q13308-2	Isoform 2 of Inactive tyrosine-protein kinase 7	6	0.324**
14	2854	46	A8K2 × 8	cDNA FLJ78433	25	0.523*
15	226	12.8	Q9ULX6	A-kinase anchor protein 8-like	6	0.612*
16	704	30.3	Q6FHK7	PSME3 protein	7	0.521*
17	479	25.4	A8K878	cDNA FLJ77177	4	0.282**
18	612	36	P84022	Smad3	5	0.233**
19	532	20.1	M0QY97	Zinc finger CCCH domain-containing protein 4	13	0.619*
20	316	22.2	Q7Z417	Nuclear fragile X mental retardation-interacting protein 2	7	0.518*
21	117	25.4	Q9UHN6	Transmembrane protein 2	5	0.317**
22	274	14.6	B4DNN4	Ribonucleoside-diphosphate reductase	9	0.516*
23	172	21.9	Q12846	Syntaxin-4	5	0.415*
24	139	25.8	H0Y5K5	Endoplasmic reticulum-Golgi intermediate compartment protein 3	4	0.414**
25	215	40.1	B4DGU4	Catenin beta-1	5	0.446**
26	287	32.7	K7EPB2	cAMP-dependent protein kinase type I-alpha regulatory subunit	9	0.332**
27	131	16.1	B2WTI3	Bifunctional arginine demethylase and lysyl-hydroxylase JMJD6	4	0.632*
28	260	15	B3KN49	cDNA FLJ13562 fis, clone PLACE1008080	5	0.211**
29	269	17.7	B3KSG9	cDNA FLJ36188 fis, clone TESTI2027179	5	0.441**
30	519	27.1	P46013	Antigen KI-67	20	0.281**
31	219	32.9	Q96A35	39S ribosomal protein L24, mitochondrial	6	0.181**
32	445	21.6	B7Z591	Transmembrane and coiled-coil domains 1, isoform CRA_a	4	0.409**
33	457	17.9	Q9NYF8-2	Isoform 2 of Bcl-2-associated transcription factor 1	11	0.338**
34	222	25.8	Q9UNK0	Syntaxin-8	6	0.467*
35	247	21.5	Q9BYK8	Helicase with zinc finger domain 2	4	0.607*
36	156	19.7	Q6LEU0	STX12 protein	4	0.557*
37	164	29.1	B2R6J0	Homo sapiens SRY (sex determining region Y)-box 2 (SOX2)	4	0.204**
38	394	16.7	Q92896-2	Isoform 2 of Golgi apparatus protein 1	19	0.304*
39	226	15.8	B4DRG7	Condensin complex subunit	10	0.514*
40	161	21.4	Q9BXK1	Krueppel-like factor 16	4	0.353*
41	281	17.4	Q8NFC6	Biorientation of chromosomes in cell division protein 1-like 1	4	0.409*
42	118	24.8	P17301	Integrin alpha-2	5	0.322**
43	457	22.9	Q15796-2	Smad2	6	0.201**
44	845	36.8	P61586	Transforming protein RhoA	6	0.374*
45	124	15.7	Q15628	Tumor necrosis factor receptor type 1-associated DEATH domain protein	4	0.26**
46	437	19.5	Q01650	Large neutral amino acids transporter small subunit 1	4	0.201*
47	718	22.9	Q86U75	Dihydropyrimidinase-like 2	10	0.244*
48	895	19.3	H3BUX2	Cytochrome b5 type B	4	0.508*
49	674	43.9	H0YKC5	Deoxyuridine 5~-triphosphate nucleotidohydrolase, mitochondrial	7	0.299*
50	355	27.2	P62906	60S ribosomal protein L10a	5	0.633*
51	287	14.5	B3KM90	cDNA FLJ10529 fis, clone NT2RP2000965	8	0.672*
52	134	17.9	Q8NCC3	Group XV phospholipase A2	4	0.495*
53	148	19.8	Q3LIB1	Putative uncharacterized protein Nbla00445	8	0.612*
54	163	20.4	O43752	Syntaxin-6	5	0.679*
55	1445	53.8	P04083	Annexin A1	15	0.586*
56	507	29.2	P20645	Cation-dependent mannose-6-phosphate receptor	7	0.385**
57	349	41.9	P60520	Gamma-aminobutyric acid receptor-associated protein-like 2	6	0.283**
58	1061	47.1	Q6FI35	Proliferating cell nuclear antigen	11	0.381**
59	156	33.4	B2RMQ4	Cytoskeleton associated protein 2	4	0.607*
60	192	16.5	G3V5T9	Cyclin-dependent kinase 2	5	0.633*
61	263	37.9	Q6IAA8	Ragulator complex protein LAMTOR1	4	0.576*
62	150	18.1	B4DJI2	cDNA FLJ53342	4	0.624*
63	108	22	B2R7M1	cDNA, FLJ93507	4	0.376*
64	140	17.3	H0Y3T6	45 kDa calcium-binding protein	4	0.558*
65	147	27.4	F8VX04	Sodium-coupled neutral amino acid transporter 1	4	0.471*
66	1103	54.2	B4DJP7	Small nuclear ribonucleoprotein Sm D3	5	0.277**
67	731	26.1	O94925-3	Isoform 3 of Glutaminase kidney isoform, mitochondrial	13	0.167**
68	178	35.3	P51151	Ras-related protein Rab-9A	5	0.267**
69	197	20.7	P15529-16	Isoform 3 of Membrane cofactor protein	4	0.566*
70	125	18.9	P46087-4	Isoform 4 of Putative ribosomal RNA methyltransferase NOP2	6	0.162**
71	268	23.6	D6W4Z6	HCG23833, isoform CRA_b	4	0.654*
72	271	27.9	B7ZM24	SLC12A2 protein	9	0.64**
73	583	42.1	P36897.1	TGF-beta receptor type-1	4	0.335**
74	321	20.2	P98172	Ephrin-B1	5	0.539*
75	192	25.3	B7Z5A7	cDNA FLJ57557	4	0.454*
76	186	39.4	B4E324	cDNA FLJ60397	4	0.552*
77	200	15.1	Q9H5V8-2	Isoform 2 of CUB domain-containing protein 1	10	0.648*
78	249	18.6	Q96T88-2	Isoform 2 of E3 ubiquitin-protein ligase UHRF1	6	0.145**
79	241	35.9	B2R7 × 3	cDNA, FLJ93645	4	0.245**
80	191	22.6	P54709	Sodium/potassium-transporting ATPase subunit beta-3	6	0.544*
81	119	18	O14672	Disintegrin and metalloproteinase domain-containing protein 10	6	0.342**
82	257	18	B3KXC3	Ferritin	5	0.557*
83	236	51.1	K7EJT5	60S ribosomal protein L22	6	0.544*
84	278	14.9	B7Z4 × 6	cDNA FLJ51012, highly similar to Plasminogen activator inhibitor 1	5	0.339**
85	1951	38.6	Q9NR30	Nucleolar RNA helicase 2	25	0.445*
86	175	15.4	B2RAK1	cDNA, FLJ94965	11	0.537*
87	209	26.9	B4DMR3	cDNA FLJ51896, highly similar to Glia-derived nexin	8	0.235**
88	332	18.6	Q53G91	Solute carrier family 16, member 3 variant (Fragment)	4	0.334*
89	130	16.3	Q5U8S2	Syntaxin 10	4	0.233**
90	136	18.2	Q9UNE7	E3 ubiquitin-protein ligase CHIP	5	0.402*
91	385	26.5	Q7Z4F3	Caveolin	4	0.471*
92	130	18.2	A8KAQ6	cDNA FLJ76490	4	0.322*
93	2202	38.5	B4DMF5	Glutamate dehydrogenase	16	0.207**
94	143	22.7	B2R6P4	cDNA, FLJ93048	4	0.113**
95	1581	68.6	P51149	Ras-related protein Rab-7a	13	0.246**
96	257	15.9	H3BRB3	Kinesin-like protein KIF22	4	0.517*
97	277	18.2	P81605-2	Isoform 2 of Dermcidin	4	0.606*
98	263	19.1	Q8N353	TMEM106B protein	5	0.399*
99	1189	34.5	Q53G71	Calreticulin variant	11	0.601*
100	395	20.9	Q13217	DnaJ homolog subfamily C member 3	5	0.374*
101	126	17.4	B2RE34	cDNA, FLJ96901	4	0.442*
102	174	26	Q53GY1	BCL2-associated athanogene 3 variant	4	0.501*
103	200	38	Q9NQW6	Actin-binding protein anillin	8	0.695*
104	110	27.9	A8K3S3	cDNA FLJ75664	5	0.326*
105	326	18.1	A8K201	cDNA FLJ75605	4	0.425*
106	115	17.9	A8K274	cDNA FLJ78227	4	0.689*
107	438	25.8	Q9NQ29-2	Isoform 2 of Putative RNA-binding protein Luc7-like 1	8	0.686*
108	20862	51.2	Q15149-4	Isoform 4 of Plectin	20	0.686*
109	164	27.5	O00161	Synaptosomal-associated protein 23	4	0.68*
110	457	18.5	Q59EZ3	Insulin-like growth factor 2 receptor variant	25	0.678*
111	362	18	A8MXZ4	G-protein-coupled receptor family C group 5 member C	5	0.673*
112	682	14.9	B3KRY3	cDNA FLJ35079 fis, clone PLACE6005283	6	0.398**
113	120	26.3	B4DN85	E3 ubiquitin-protein ligase	4	0.595*
114	108	10.8	O15269	Serine palmitoyltransferase 1	5	0.67*
115	382	24.2	B4DL49	cDNA FLJ58073, moderately similar to Cathepsin B	5	0.67*
116	139	16	H0YDJ9	CD81 antigen	4	0.657*
117	522	32.4	B4DKJ4	cDNA FLJ57738	4	0.232**
118	149	25.2	B5BU32	Thymidine kinase	5	0.652*
119	122	33.8	O75976	Carboxypeptidase D	4	0.332*
120	618	19.3	P29317	Ephrin type-A receptor 2	11	0.651*
121	117	24.1	D6RAR4	Hepatocyte growth factor activator	4	0.451*
122	198	32.6	G3V3D1	Epididymal secretory protein E1	6	0.643*
123	286	30.7	C0JYY2	Apolipoprotein B	4	0.64*
124	114	29	F5H569	V-type proton ATPase 116 kDa subunit a isoform 1	6	0.276**
125	178	31.7	B4E1K0	Kinesin-like protein KIF23	4	0.633*
126	156	51.4	Q53HU8	vimentin	5	0.413*
127	125	34.5	Q14118	Dystroglycan	4	0.614*
128	128	16.2	C1K3N4	Tumor necrosis factor receptor superfamily member 10a	4	0.592*
129	165	32.6	Q13501-2	Isoform 2 of Sequestosome-1	4	0.58*
130	290	26.8	F5GZY0	Amyloid-like protein 2	4	0.576*
131	410	26.2	B4DJQ8	cDNA FLJ5569	8	0.174**
132	1790	32.8	P11387	DNA topoisomerase 1	26	0.044**
133	186	17.3	B2R686	Trans-golgi network protein 2, isoform CRA_a	4	0.542*
134	191	30.7	H0Y8A7	NEDD4 family-interacting protein 2	4	0.331**
135	112	27.7	P62266	40S ribosomal protein S23	5	0.53*
136	159	33.4	B3KMB6	cDNA FLJ10642 fis, clone NT2RP2005752	7	0.53*
137	175	25	B4DSG5	cDNA FLJ56149	5	0.525*
138	893	24.8	Q71UA6	Neutral amino acid transporter	10	0.499*
139	179	15.8	A8K6H9	cDNA FLJ75876	5	0.486*
140	198	28.6	Q9NRX5	Serine incorporator 1	4	0.442*
141	125	18.8	B4DIB1	cDNA FLJ55065	5	0.427*
142	137	17.3	P37173-2	Isoform 2 of TGF-beta receptor type-2	4	0.349*
143	753	25.1	P55010	Eukaryotic translation initiation factor 5	9	0.315*
144	121	35.7	P14174	Macrophage migration inhibitory factor	4	0.299**
145	325	24.1	E7EQY1	Protein FAM136A	5	0.432*
146	124	19.6	Q9NY27	Serine/threonine-protein phosphatase 4 regulatory subunit 2	4	0.411*
147	900	24.4	P62306	Small nuclear ribonucleoprotein F	4	0.558*
148	256	19.8	C9JEH3	Angio-associated migratory cell protein	7	0.512**

^a^Regulations (fold-changes) of differentially expressed proteins in MDA-MB-231 cells (metapristone-treatment versus control). *P < 0.05; **P < 0.01.

**Table 3:** Pathway analysis of the DEPs obtained from the iTRAQ analysis.

Pathway description	Count	*P*-value
Metabolic pathways	81	5.21E-11
RNA transport	74	1.13E-10
Endocytosis	58	1.49E-10
Oxidative phosphorylation	56	2.83E-09
Apoptosis	53	5.78E-09
Focal adhesion	48	1.18E-08
MAPK signaling pathway	36	5.62E-08
Regulation of actin cytoskeleton	35	8.21E-08
GnRH signaling pathway	33	1.91E-07
B cell receptor signaling pathway	31	2.05E-07
Calcium signaling pathway	30	1.13E-06
Chemokine signaling pathway	28	3.61E-06
NF-kappa B signaling pathwy	27	4.13E-06
Peroxisome	27	1.70E-05
T cell receptor signaling pathway	25	2.18E-05
ErbB signaling pathway	23	3.62E-05
Neurotrophin signaling pathway	23	4.21E-05
Toll-like receptor signaling pathway	23	4.36E-05
Jak-STAT signaling pathway	23	6.99E-05
Insulin signaling pathway	20	0.000134
Notch signaling pathway	21	0.000313
ECM-receptor interaction	19	0.000397
mTOR signaling pathway	19	0.000724
p53 signaling pathway	17	0.000797
TGF-beta signaling pathway	17	0.000913
VEGF signaling pathway	16	0.001033
PPAR signaling pathway	15	0.001334
Adherens junction	13	0.001427
Wnt signaling pathway	12	0.003628
Cell adhesion molecules (CAMs)	11	0.003316
Drug metabolism-cytochrome P450	8	0.004733
ABC transporters	7	0.007124
Regulation of autophagy	6	0.008114

There were 249 pathways revealed. Among them, the following 33 signaling pathways were significant (*P* < 0.01).
